# Abolishing spontaneous epileptiform activity in human brain tissue through AMPA receptor inhibition

**DOI:** 10.1002/acn3.51030

**Published:** 2020-05-19

**Authors:** Sukhvir K. Wright, Max A. Wilson, Richard Walsh, William B. Lo, Nilesh Mundil, Shakti Agrawal, Sunny Philip, Stefano Seri, Stuart D. Greenhill, Gavin L. Woodhall

**Affiliations:** ^1^ Aston Neuroscience Institute School of Life and Health Sciences Aston University Birmingham UK; ^2^ Department of Paediatric Neurology The Birmingham Women’s and Children's Hospital NHS Foundation Trust Birmingham UK; ^3^ Department of Paediatric Neurosurgery The Birmingham Women’s and Children's Hospital NHS Foundation Trust Birmingham UK; ^4^ Department of Clinical Neurophysiology The Birmingham Women’s and Children's Hospital, NHS Foundation Trust Birmingham UK

## Abstract

**Objective:**

The amino‐3‐hydroxy‐5‐methyl‐4‐isoxazolepropionic acid receptor (AMPAR) is increasingly recognized as a therapeutic target in drug‐refractory pediatric epilepsy. Perampanel (PER) is a non‐competitive AMPAR antagonist, and pre‐clinical studies have shown the AMPAR‐mediated anticonvulsant effects of decanoic acid (DEC), a major medium‐chain fatty acid provided in the medium‐chain triglyceride ketogenic diet.

**Methods:**

Using brain tissue resected from children with intractable epilepsy, we recorded the effects of PER and DEC in vitro*.*

**Results:**

We found resected pediatric epilepsy tissue exhibits spontaneous epileptic activity in vitro, and showed that DEC and PER inhibit this epileptiform activity in local field potential recordings as well as excitatory synaptic transmission.

**Interpretation:**

This study confirms AMPAR antagonists inhibit epileptiform discharges in brain tissue resected in a wide range of pediatric epilepsies.

## Introduction

Molecular targets for pediatric epilepsy treatment have largely centered on voltage‐gated sodium and calcium channels. The recent introduction of Perampanel (PER), a non‐competitive amino‐3‐hydroxy‐5‐methyl‐4‐isoxazolepropionic acid receptor (AMPAR) antagonist, has introduced the concept of AMPAR modulation as an effective anticonvulsant strategy.^1^, ^2^ Similarly, recent studies in epilepsy rodent models have demonstrated the anti‐epileptic action of decanoic acid (DEC), a major medium‐chain fatty acid provided in the medium‐chain triglyceride (MCT) ketogenic diet (KD), is mediated through direct and selective inhibition of AMPARs.^3^, ^4^

Human tissue samples provide a valuable tool for pre‐clinical drug screening and mechanism‐of‐action epilepsy studies.^5^ This is particularly useful in pediatrics, where few anticonvulsant drug trials include children and adult study results are often extrapolated to children with pediatricians prescribing “off‐label”.^6^ In most human brain tissue preparations, epileptiform activity is induced through manipulation of artificial cerebrospinal fluid (aCSF) magnesium or potassium or by adding pro‐convulsant agents (e.g. 4‐aminopyridine).^7^, ^8^, ^9^, ^10^ We have found pediatric human brain tissue to be hyperexcitable and have recorded *spontaneous* epileptic activity. We show that antagonism of AMPAR has a profound anticonvulsant effect in tissue derived from a spectrum of difficult‐to‐treat seizure syndromes.

## Methods

### Human tissue collection and slice preparation

Human tissue was obtained with informed consent from pediatric patients undergoing epilepsy surgery at Birmingham Children’s Hospital. Ethical approval was obtained from the Black Country Local Ethics Committee (10/H1202/23; 30 April 2010), and from Aston University’s ethics committee (Project 308 cellular studies in epilepsy) and through the Research and Development Department at Birmingham Children’s Hospital (IRAS ID12287). Specimens were resected intraoperatively with minimal traumatic tissue damage, and minimal use of electrocautery. For transport to the laboratory, samples were transferred immediately to ice‐cold choline‐based artificial cerebrospinal fluid (aCSF) standardized for use in human tissue experiments^8^, ^9^, ^10^, ^11^ containing in mmol/L: 110 choline chloride, 26 NaHCO_3_, 10 D‐glucose, 11.6 ascorbic acid, 7 MgCl_2_, 3.1 sodium pyruvate, 2.5 KCl, 1.25 NaH_2_PO_4_, and 0.5 CaCl_2_ with added 0.04 indomethacin and 0.3 uric acid for neuroprotection, and bubbled with carbogen (95% O_2_, 5% CO_2_). For slice storage and experiments, aCSF containing (in mmol/L): 125 NaCl, 3 KCl, 1.6 MgSO_4_, 1.25 NaH_2_PO_4_, 26 NaHCO_3_, 2 CaCl_2_, 10 Glucose, was used.

### Local field potential (LFP) recordings

About 450 µm thick brain slices were prepared and stored and recordings made as previously reported.^12^ Epileptiform events were classified as activity displaying an amplitude fourfold greater than the root mean square baseline amplitude, providing the event count, while the time difference between these events provided the interevent interval (IEI). Statistical analysis was conducted using Prism 8. Measurements expressed as median (M), interquartile range (Q1–Q3) and min–max values.

### Whole‐cell patch‐clamp (WCPC) recordings

Whole‐cell recordings were made using standard techniques.^13^ Electrodes were filled with an internal solution containing (in mmol/L): 100 CsCl, 40 HEPES, 1 Qx‐314, 0.6 EGTA, 5 MgCl_2_, 10 TEA‐Cl, 4 ATP‐Na, 0.3 GTP‐Na (titrated with CsOH to pH 7.25) at 290–295 mOsm for IPSCs. Also included in the electrode was 1–3 mmol/L 1 IEM 1460, which blocks ionotropic glutamate receptors from inside the cell.^13^ For EPSCs the internal solution contained (in mmol/L): 100 Cs‐gluconate, 40 HEPES, 1 Qx‐314, 0.6 EGTA, 2 NaCl, 5 Mg‐gluconate, 5 TEA‐Cl, 10 Phospho‐Creatinine, 4 ATP‐Na, 0.3 GTP‐Na (titrated with CsOH to pH 7.3) at 285 mOsm. The EPSCs and IPSCs were recorded as apparent inward currents at −70 mV using Axopatch 200B amplifier (Molecular Devices, San Jose, CA, USA). Signals were low‐pass filtered at 5 kHz with an 8‐pole Bessel filter and digitized at 10 kHz using a Digidata 1440A and pClamp software (Molecular Devices). Data were analyzed using Axograph and Prism 8. Measurements are expressed as mean median ± SEM.

### Drugs

DEC (Sigma, Dorset, UK) and PER (Eisai, Hatfield, UK) were prepared as 1 M stock using dimethyl sulfoxide.

## Results

### Patient data

Brain tissue was obtained from 16 patients (F:M 9:7), median age 10.5 years (range 3–18 years). Surgical procedures included temporal resection (seven), hemispherectomy (five), occipital lobectomy (one), and frontal resection (three). One patient was on the modified KD pre‐surgery (Table 1).

**Table 1:** Patient data.

Patient	Operation	Medication	Histology
1	Right frontal disconnection and resection	Modified KD Sodium valproate Vigabatrin	No definite abnormal pathology
2	Left temporal lobectomy	Topiramate Carbamazepine	Gliosis
3	Right temporal lobectomy	Lamotrigine	Gliosis, in keeping with secondary changes associated with epilepsy
4	Hemispherotomy	Clonazepam Carbamazepine Levetiracetam	Gliosis
5	Left temporal lobectomy and hippocampectomy	Clobazam Levetiracetam Lamotrigine	Hippocampal sclerosis
6	Left temporal lobectomy	Lamotrigine	Hippocampal sclerosis
7	Hemispherotomy	Oxcarbazepine Levetiracetam	Gliosis, in keeping with secondary changes associated with epilepsy
8	Left temporal lobectomy	Topiramate Carbamazepine	Low grade glioneuronal neoplasm
9	Left temporal lobectomy	Carbamazepine	Hippocampal sclerosis
10	Occipital lobectomy	Lamotrigine Sodium valproate	Cortical damage
11	Frontal resection	Topiramate Clobazam	Focal cortical dysplasia Type IIB
12	Frontal resection	Carbamazepine Brivacetam	Focal cortical dysplasia Type IA
13	Temporal lobectomy	Levetiracetam Carbamezapine	Mesial temporal sclerosis
14	Hemispherotomy	Topiramate Carbamazepine Clobazam	Focal cortical dysplasia
15	Hemispherotomy	Lamotrigine Carbamezapine	Rasmussens encephalitis
16	Hemispherotomy	Levetiracetam Caarbamezapine	Polymicrogyria

### DEC reduces spontaneous epileptiform activity in paediatric epilepsy tissue in vitro

**Figure 1:**
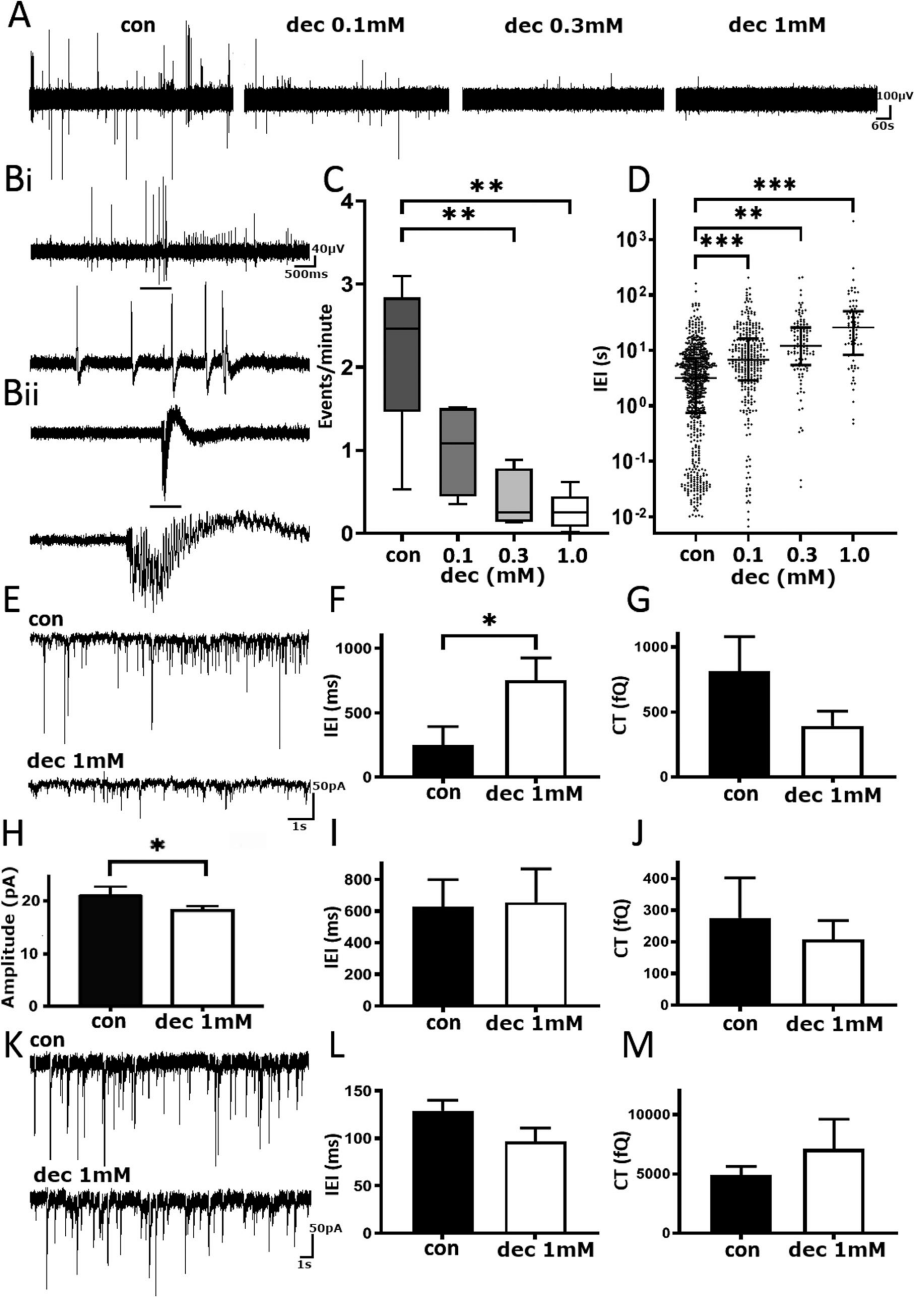
Decanoic acid reduces spontaneous epileptiform activity in pediatric epilepsy tissue samples. (A) Example trace of local field potential recording before and after consecutive addition of DEC. Scale bar 100 µV versus 60 sec. (B) Two examples of spontaneous epileptiform activity from separate pediatric human tissue recordings. Black bar denotes zoomed in section of events. Scale bars (top) 40 µV versus 500 msec, (bottom) 100 µV versus 2 sec. (C) Pooled events per minute under various conditions. (D) The interevent interval in different concentrations of DEC. (E) Example trace of sEPSCs from pediatric human tissue before (top) and after (bottom) treatment with 1 mmol/L DEC. Scale bar 50 pA versus 1 sec. (F) Pooled mean‐median inter‐event interval in 1 mmol/L DEC. (G) Pooled sEPSC charge transfer in 1 mmol/L DEC. (H) Pooled mean‐medium amplitude of sEPSCs in 1 mmol/L DEC. (I) Pooled inter‐event interval of mEPSCs with 1 mmol/L DEC. (J) Pooled charge transfer of mEPSCs in 1 mmol/L DEC. (K) Example traces of mIPSC recordings pre and post addition of 1 mmol/L DEC. Scale bar 50 pA versus 1 sec. (L) Pooled interevent interval of mIPSCs in control and 1 mmol/L DEC. (M) Pooled charge transfer of mIPSCS after addition of 1 mmol/L DEC. DEC, decanoic acid. *P ≤ 0.05; **P ≤ 0.01; ***P ≤ 0.001.

LFP recordings were assessed for spontaneous epileptiform activity (Fig. 1B) before addition of DEC at increasing concentrations (Fig. 1A). DEC significantly reduced the frequency of epileptiform events per minute compared to control conditions (control [M = 2.46, Q1–Q3 = 1.47–2.84, min–max = 0.53–3.10; *n* = 5] vs. 300 µmol/L [M = 0.25, Q1–Q3 = 0.14–0.78, min–max = 0.13–0.88; *n* = 5, *P* < 0.05] and 1 mmol/L concentrations [M = 0.25, Q1–Q3 = 0.08–0.45, min–max = 0.02–0.62; *n* = 5, *P* < 0.05]; Friedman test with Dunn’s post‐test; Fig. 1C). The IEI of epileptic activity increased significantly with all concentrations of DEC (Control [M = 3.18 sec, Q1–Q3 = 0.75–7.33 sec; *n* = 5] vs. 100 µmol/L [M = 6.76 sec, Q1–Q3 = 2.87–16.40 sec; *n* = 5, *P* < 0.005], 300 µmol/L [M = 12.12 sec, Q1–Q3 = 5.44–26.01 sec; *n* = 5, *P* < 0.005] and 1 mmol/L [M = 26.11 sec, Q1–Q3 = 8.29–51.27 sec; *n* = 5, *P* < 0.005]; Kruskal–Wallis with Dunn’s post‐test; Fig. 1D).

### DEC inhibits excitatory but not inhibitory postsynaptic currents

The effects of DEC on synaptic activity were investigated using the WCPC technique to assess spontaneous excitatory and inhibitory postsynaptic currents (sEPSCs/sIPSCs; Fig. 1E–M). As Figure 1F shows, application of 1 mmol/L DEC significantly increased the IEI (248 ± 145 vs. 752 ± 175 msec, *n* = 6 cells, *P* = 0.03) with no change in the amplitude of sEPSCs (19.8 ± 1.9 vs. 17.4 ± 1.2 pA, *n* = 6, *P* = 0.1), or charge transfer (Fig. 1G; 641 ± 265 vs. 350 ± 114 fC, *n* = 6, *P* = 0.5). We next tested the location of the DEC effects by testing the effect of DEC in the presence of tetrodotoxin (TTX, 1 µmol/L). As Figure 1H shows, after addition of TTX there was a significant reduction of amplitude (20.9 ± 1.9 vs. 18.2 ± 0.8 pA, *n* = 6 cells, *P* = 0.03), but no significant change in the IEI of mEPSCs (Fig. 1I; 627 ± 172 vs. 656.5 ± 211 msec, *n* = 6 cells, *P* = ns) or charge transfer (Fig. 1J; 192 ± 127 vs. 195 ± 60 fC, *n* = 6, *P* = 0.5) with 1 mmol/L DEC, suggesting DEC may act on postsynaptic AMPARs.

Since inhibitory interneurons are subject to glutamatergic drive, any change in AMPAR activity is likely to depress gamma‐aminobutyric acid (GABA) release and, indeed, we have seen this in recordings where PER is applied. It seems unlikely, however, that DEC would have any direct effect on GABA release. To investigate the actions of DEC on isolated inhibitory synaptic currents, we used whole‐cell patch electrodes filled with the ionotropic glutamate receptor channel blocker, IEM‐1460, at 1.5 mmol/L. Under these conditions, in which AMPARs were blocked from inside the recorded cell, we recorded miniature IPSCs (mIPSCs) from principal neurons in the presence of TTX 1 µmol/L (Fig. 1K). Analysis of pooled data revealed no significant change in amplitude, (28.9 ± 2.5 vs. 30.1 ± 2.2 pA, *n* = 5, *P* = ns), IEI of recorded mIPSCS after addition of 1 mmol/L DEC (Fig. 1L; 129.3 ± 10.9 vs. 96.6 ± 14.3 msec, *n* = 5, *P* = ns), or charge transfer (Fig. 1M; 4912 ± 728 vs. 5751 ± 2503 fQ, *n* = 5, *P* = ns).

### PER abolishes spontaneous epileptiform activity in pediatric epilepsy tissue in vitro

**Figure 2:**
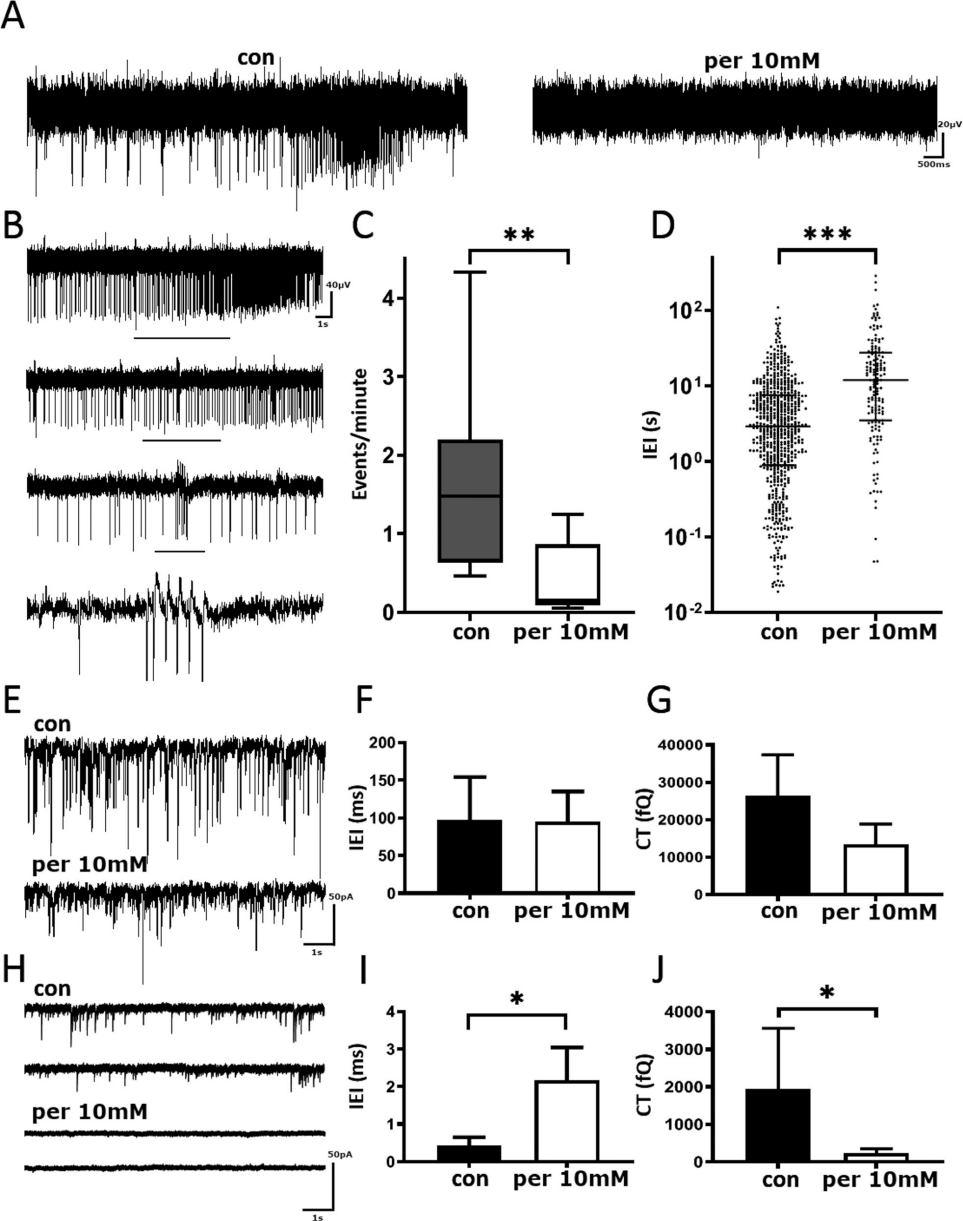
Perampanel reduces spontaneous epileptiform activity in pediatric epilepsy tissue samples. (A) Example trace of local field potential recording before and after the addition of 10 µmol/L PER. Scale bar 20 µV versus 500 msec. (B) Example of spontaneous epileptiform activity. Black bar denotes zoomed in section of events. Scale bar 40 µV versus 1 sec. (C) Number of events per minute before and after the addition of 10 µmol/L PER. (D) Pooled interevent interval before and after application of 10 µmol/L PER. (E) Example trace of sIPSCs from pediatric human tissue before (top) and after (bottom) treatment with 10 µmol/L of Perampanel. Scale bar 50 pA versus 1 sec. (F) Pooled sIPSC inter‐event interval before and after application of 10 µmol/L PER (G) Pooled charge transfer of sIPSCs before and after application of 10 µmol/L PER. (H) Example trace of sEPSCs from pediatric human tissue before (top) and after (bottom) treatment with perampanel. Scale bar 50 pA versus 1 sec. (I) Pooled inter‐event interval before and after application of 10 µmol/L PER. (J) Pooled charge transfer before and after application of 10 µmol/L PER. PER, perampanel. *P ≤ 0.05; **P ≤ 0.01; ***P ≤ 0.001.

LFP recordings were assessed for spontaneous epileptiform activity (Fig. 2B) before addition of PER (Fig. 2A). PER significantly reduced the number of epileptiform events per minute compared to control conditions (control [M = 1.48, Q1–Q3 = 0.63–2.20, min–max = 0.47–4.33; *n* = 7] vs. PER 10 µmol/L [M = 0.35, Q1–Q3 = 0.13–0.83, min–max = 0.05–0.87]; *n* = 7, *P* < 0.05; Wilcoxon test Fig. 2C). Application of PER also increased the IEI (Fig. 2D; control [M = 2.89 sec, Q1–Q3 = 0.89–7.51 sec; *n* = 7] vs. 10 µmol/L PER [M = 12.01 sec, Q1–Q3 = 3.50–27.48 sec; *n* = 7], *P* < 0.005).

### PER inhibits excitatory but not inhibitory postsynaptic currents

To gain a better understanding of PER’s ability to abolish spontaneous epileptiform activity, recordings of neurotransmitter release were conducted (Fig. 2E–J). Recordings revealed that principal neurons received GABAergic IPSCs with a mean median IEI of 96.99 ± 23.23 msec (Fig. 2F) and a mean median amplitude of 36.28 ± 8.33 pA. In the presence of PER (10 µmol/L) there was no significant change in IEI (Fig. 2F; 88.93 ± 16.55 msec, *n* = 6, *P* = ns), amplitude (25.19 ± 3.82 pA, *P* > 0.05) or charge transfer (Fig. 2G; 16.46 ± 10.91 vs. 10.16 ± 0.54 pC, *n* = 6, *P* = 0.1). When we recorded sEPSCs (Fig. 2H), IEI was significantly increased from 173 ± 217 to 2361 ± 873 msec (*P* = 0.03) in the presence of PER (Fig. 2I). The effect of PER on amplitude did not reach statistical significance (19.87 ± 4.1 to 10.83 ± 2.92 pA; *P* = 0.06), but the charge transfer was significantly reduced (Fig. 2J; 341 ± 1622 vs. 169 ± 115 fC, *n* = 6, *P* = 0.03).

## Discussion

This study confirms that AMPAR inhibition, either by DEC or PER, is effective in abolishing spontaneous epileptiform activity in human tissue from drug‐resistant pediatric epilepsy patients through a direct reduction in excitatory neurotransmission.

DEC, a major constituent of the MCT KD, has previously been shown to have an anticonvulsant action in acute in vitro rat hippocampal slice models of epileptiform activity, acting through modulation of excitatory neurotransmission.^3^, ^4^, ^5^ In our human tissue LFP recordings, the anticonvulsant effects of DEC were clearly demonstrated at the 300 µmol/L concentration, consistent with reported therapeutic pediatric plasma concentrations.^14^, ^15^ In WCPC experiments, the anticonvulsant mechanism was shown to be likely through the reduction of post‐synaptic excitatory neurotransmission via AMPARs.

Similar effects were seen with PER, via a reduction in the frequency of EPSCs and overall charge transfer. These effects were observed in tissue from a wide array of epilepsy syndromes, and underline the importance of the AMPAR in understanding the development and treatment of the epilepsies. Like many aspects of epilepsy, it is not a simple case of ‘too much’ AMPAR activity leading to seizures, for example, in anti‐AMPAR autoimmune encephalitis, antibodies generated against AMPAR epitopes lead to AMPAR internalization, thereby reducing excitatory drive while still causing temporal lobe seizures in man.^16^, ^17^ Similarly, recent work in a rat model of chronic TLE in our laboratory suggests that seizures induce a profound loss of AMPAR expression in vulnerable networks.^12^ Hence, AMPARs would appear to play both causative and compensatory roles in seizures and epileptogenesis.

Currently, the management for children with drug‐resistant epilepsy includes referral to an epilepsy surgery unit for assessment of suitability for resective surgery. Researchers have attempted to explore the role of the KD in improving seizure outcomes in epilepsy surgery patients with Focal Cortical Dysplasia Type II (the EDIBLE research study: www.edible.org.uk). While studies in human brain tissue do not provide a substitute for clinical trials in children, this preclinical study suggests that the MCT KD or PER may be effective in reducing seizure burden pre‐operatively for these patients. Indeed, a synergistic therapeutic effect of DEC and PER was recently demonstrated in a study using in vitro epilepsy animal models and adult human tissue from brain tumor patients.^4^

One criticism of human epileptic tissue research is the lack of “control” tissue.^18^, ^19^ For obvious ethical reasons, we cannot and will never have the opportunity to obtain pediatric “normal” brain tissue for the ideal control. However, this should not prevent the use of human tissue as an invaluable resource to study the true pathophysiological changes in pediatric drug‐resistant epilepsy. Recent studies in human brain tissue have demonstrated the presence of extensive species‐specific differences in neuron types and properties as compared to the rodent brain.^20^, ^21^ A clear example of this is the expression of 5HT3 receptors, which are present on excitatory cells throughout the human forebrain, but expressed only on inhibitory GABA cells in rodent brain.^22^ Therefore, the use of human epileptic brain tissue could provide novel insights to complement preclinical animal model studies. This is of the particular importance of the testing of treatments in medically refractory epilepsy, as few animal models recapitulate fully the diversity of seizure types, localization and etiology of the patients seen in the clinic. In pediatric epilepsy research, slice preparations from resected tissue are extremely valuable and rare, and provide a unique substrate for testing novel and approved compounds in pharmacoresistant epilepsy.

In summary, this study shows the potential application of human tissue samples for epilepsy research and drug development. The heterogeneity of the human tissue samples is a true reflection of the variable etiology of refractory epilepsy and should be considered a strength of the human tissue approach to pediatric epilepsy research.

## Author Contributions

SKW, GLW, and SDG contributed to the conception and design of the study. SKW, GLW, SDG, SS, MAW, ARW, WBL, NM, SA, SP contributed to the acquisition and analysis of data. SKW, GLW, SDG, and MAW contribute to the drafting of the manuscript and figures.

## Conflicts of Interests

SP has received speaker’s fees from Eisai, LivaNova, Novartis and Zogenix. GLW and SDG have been funded by GW Pharma for work unrelated to this paper.
